# miR-375 Regulation of SSTR2 Expression in Corticotroph Pituitary Cells: Somatostatin Receptor Ligands Effects

**DOI:** 10.1210/endocr/bqaf107

**Published:** 2025-06-09

**Authors:** Claudia Pivonello, Roberta Patalano, Mariarosaria Negri, Donatella Treppiedi, Erika Peverelli, Feliciana Amatrudo, Donatella Paola Provvisiero, Chiara Simeoli, Nicola Di Paola, Angelica Larocca, Erminio Massimo Crescenzo, Giovanna Mantovani, Annamaria Colao, Rosario Pivonello

**Affiliations:** Dipartimento di Sanità Pubblica, Università Degli Studi di Napoli “Federico II”, Naples 80131, Italy; Dipartimento di Medicina Clinica e Chirurgia, Sezione di Endocrinologia, Diabetologia, Andrologia e Nutrizione, Università Degli Studi di Napoli “Federico II”, Naples 80131, Italy; Dipartimento di Medicina Clinica e Chirurgia, Sezione di Endocrinologia, Diabetologia, Andrologia e Nutrizione, Università Degli Studi di Napoli “Federico II”, Naples 80131, Italy; Dipartimento di Psicologia e Scienze Della Salute, Università Telematica Pegaso, Naples 80143, Italy; Endocrinology Unit, Fondazione Istituto di Ricovero e Cura a Carattere Scientifico (IRCCS) Ca’ Granda Ospedale Maggiore Policlinico Milan 20122, Italy; Endocrinology Unit, Fondazione Istituto di Ricovero e Cura a Carattere Scientifico (IRCCS) Ca’ Granda Ospedale Maggiore Policlinico Milan 20122, Italy; Department of Clinical Sciences and Community Health, University of Milan, Milan 20122, Italy; Dipartimento di Medicina Clinica e Chirurgia, Sezione di Endocrinologia, Diabetologia, Andrologia e Nutrizione, Università Degli Studi di Napoli “Federico II”, Naples 80131, Italy; Dipartimento di Psicologia e Scienze Della Salute, Università Telematica Pegaso, Naples 80143, Italy; Dipartimento di Medicina Clinica e Chirurgia, Sezione di Endocrinologia, Diabetologia, Andrologia e Nutrizione, Università Degli Studi di Napoli “Federico II”, Naples 80131, Italy; Dipartimento di Medicina Clinica e Chirurgia, Sezione di Endocrinologia, Diabetologia, Andrologia e Nutrizione, Università Degli Studi di Napoli “Federico II”, Naples 80131, Italy; Dipartimento di Medicina Clinica e Chirurgia, Sezione di Endocrinologia, Diabetologia, Andrologia e Nutrizione, Università Degli Studi di Napoli “Federico II”, Naples 80131, Italy; Dipartimento di Medicina Clinica e Chirurgia, Sezione di Endocrinologia, Diabetologia, Andrologia e Nutrizione, Università Degli Studi di Napoli “Federico II”, Naples 80131, Italy; Dipartimento di Medicina Clinica e Chirurgia, Sezione di Endocrinologia, Diabetologia, Andrologia e Nutrizione, Università Degli Studi di Napoli “Federico II”, Naples 80131, Italy; Endocrinology Unit, Fondazione Istituto di Ricovero e Cura a Carattere Scientifico (IRCCS) Ca’ Granda Ospedale Maggiore Policlinico Milan 20122, Italy; Department of Clinical Sciences and Community Health, University of Milan, Milan 20122, Italy; Dipartimento di Medicina Clinica e Chirurgia, Sezione di Endocrinologia, Diabetologia, Andrologia e Nutrizione, Università Degli Studi di Napoli “Federico II”, Naples 80131, Italy; UNESCO Chair for Health Education and Sustainable Development, Federico II University, Naples 80131, Italy; Dipartimento di Medicina Clinica e Chirurgia, Sezione di Endocrinologia, Diabetologia, Andrologia e Nutrizione, Università Degli Studi di Napoli “Federico II”, Naples 80131, Italy; UNESCO Chair for Health Education and Sustainable Development, Federico II University, Naples 80131, Italy

**Keywords:** miR-375, somatostatin receptor type 2 (SSTR2), Cushing disease, Cushing syndrome

## Abstract

Long-term exposure to glucocorticoids (GCs) downregulates SSTR2 expression in corticotroph tumors, limiting the efficacy of octreotide (OCT) in the treatment of Cushing disease (CD). In AtT20 cells, dexamethasone (DEX) increased the expression of miR-375, which has a seed sequence for *Ssrt2*, supporting the hypothesis that excessive GC exposure can lead to epigenetic SSTR2 downregulation. The current study aims to evaluate miR-375 levels by reverse transcription quantitative polymerase chain reaction in sera from patients with CD, human corticotroph pituitary tumors, normal pituitaries, and AtT20/D16 and GH3 cells, and miR-375 impact on SSTR2 expression in AtT20/D16 and human corticotroph pituitary tumors. SSTR2 protein expression and localization were evaluated by WB and IF in AtT20/D16 and human primary cultures. Proliferation assay and flow cytometry were assessed to investigate the impact of miR-375 regulation on OCT treatment in AtT20/D16. miR-375 levels were higher in sera from patients with CD than in healthy subjects, and in human corticotroph pituitary tumors than in normal pituitaries. AtT20/D16 and GH3 exhibited an inverse expression pattern, with SSTR2 mRNA at low levels and miR-375 at high levels in AtT20/D16 and an opposite expression pattern in GH3. DEX treatment significantly reduced SSTR2 gene expression, while miR-375 inhibition significantly increased SSTR2 membranous protein expression in AtT20/D16 and primary cultures. Receptor internalization appeared stronger when OCT was combined with miR-375 inhibitor. The decreased cell proliferation induced by OCT was potentiated by miR-375 inhibition, increasing cells in early and late apoptosis, by inducing PARP, Caspase3, and ERK1/2 phosphorylation. In conclusion, SSTR2 protein expression can be epigenetically downregulated by GC-induced miR-375 expression, at least partially influencing OCT action in corticotroph pituitary tumors.

Pituitary surgery still represents the first-line treatment for patients with Cushing disease (CD) (1, 2). Somatostatin receptor (SSTR) expression in pituitary corticotroph tumors led to the clinical use of somatostatin receptor ligands (SRLs). SRLs represent pituitary-directed drugs potentially useful for the control of adrenocorticotroph hormone (ACTH) secretion, which leads to a reduction of endogenous cortisol excess, in patients with CD in whom pituitary surgery is not an option or has not been curative (1, 2). The first-generation and the second-generation SRLs have been studied in in vitro and in vivo settings; nevertheless, only the second-generation SRL pasireotide (PAS) has demonstrated a relevant effect on ACTH and cortisol production and has been approved by the regulatory authorities for the treatment of patients with CD (3). The major efficacy of PAS compared with the first-generation SRL octreotide (OCT) appears to be mainly, but not exclusively, attributed to the SSTR pattern expressed by pituitary corticotroph tumors, which predominantly express SSTR5 (4, 5). Indeed, PAS binds multiple subtypes of SSTRs, with a significantly higher binding affinity for SSTR5, whereas OCT preferentially binds with higher affinity to SSTR2 (2, 6, 7). It has been hypothesized that the lack of clinical efficacy of OCT in the control of ACTH and cortisol excess in CD may result from the suppressive effects of long-term exposure to cortisol excess on *SSTR2*, but not *SSTR5*, mRNA expression in pituitary corticotroph tumors (4, 8). This hypothesis has also been supported by the observation that the nonselective glucocorticoid (GC) receptor antagonist mifepristone (4) and the selective GC receptor antagonist relacorilant (9) were able to reverse the GC suppressive effect on *SSTR2* mRNA expression in human neuroendocrine cell lines and in the murine pituitary corticotroph tumor cell line, AtT20. Interestingly, while mifepristone simply reverted GC effects without positively affecting *SSTR2* mRNA expression in human neuroendocrine cell lines (4), the use of relacorilant was also associated with *Sstr2* mRNA overexpression by inhibiting the dexamethasone (DEX)-mediated reduction of *Sstr2* mRNA in a concentration-dependent manner in the AtT20 cell line (9). The regulation of gene expression by GCs involves various mechanisms that may lead to either activation or inhibition of target gene transcription (10). *SSTR2* gene expression is finely transcriptionally downregulated by high levels of GCs (4, 8, 11) through several different mechanisms, including competitive binding of GC receptor and stimulatory factors to regulatory DNA elements and interaction of GC receptor with negative regulatory DNA elements within gene promoters (10, 12).

In contrast, there is a dearth of investigation into the involvement of microRNA (miRNA) in modulating GC-dependent gene expression regulation. Mature miRNAs, belonging to the large family of noncoding RNAs, play an important role in the regulation of gene expression. Indeed, as epigenetic mediators, miRNAs negatively regulate the protein levels of the target genes by binding to the 3′ untranslated region or the 5′ untranslated region, gene promoter region, or the coding sequence and consequently by triggering either translational inhibition or transcript degradation (13). DEX treatment of a murine corticotroph tumor cell model, AtT20, increased miR-375 expression (14), analysis of which revealed a seed sequence for *Sstr2*, supporting the hypothesis that GCs excess might downregulate SSTR2 expression even through an epigenetic mechanism.

Given the intriguing scenario, the current study aimed to assess miR-375 circulating and pituitary tissue levels in patients with CD and to evaluate the impact of miR-375 on SSTR2 expression modulation in a strain of the murine pituitary corticotroph tumor cell line AtT20, AtT20/D16, and in 2 human corticotroph pituitary primary cell cultures, and, consequently, to assess the effect of OCT and PAS, alone and in combination with miR-375 inhibitor, on cell proliferation.

## Materials and Methods

### Study Design

In the current study, serum and tissue samples from patients with active CD were used as ex vivo models, and the AtT20/D16 cell line and 2 human primary cultures were used as in vitro models of murine and human corticotroph pituitary tumors. The study assessed (1) circulating levels of miR-375 expression in 26 serum samples from patients with active CD and 17 sex-matched and age-matched healthy subjects; (2) tissue levels of miR-375 in 6 human corticotroph pituitary tumors and 2 normal pituitary tissues; and (3) miR-375 levels in in vitro models of corticotroph and somatotroph pituitary tumors, AtT20/D16 and GH3, respectively, by reverse transcription quantitative polymerase chain reaction (RT-qPCR). Concomitantly, *Sstr2* mRNA expression was also evaluated in AtT20/D16 and GH3, and in AtT20/D16 treated with DEX 10^−8^ M, by RT-qPCR. Furthermore, the effect of miR-375 on basal SSTR2 protein expression in AtT20/D16 and cells of 2 human primary cultures of corticotroph pituitary tumors was examined after 24 hours of cell transfection with miR-375 inhibitor and mimic in a complete medium, by immunofluorescence (IF) and Western blot (WB). To demonstrate the potential impact of miR-375 on SRL activity, a proliferation assay was performed in AtT20/D16 after treatment with OCT and PAS at 10^−8^ M and 10^−7^ M alone and in combination with miR-375 inhibitor. Based on the obtained results, SSTR2 expression and cellular localization were investigated in AtT20/D16 by IF after 24 hours of cell transfection with miR-375 inhibitor and 20 minutes of OCT at 10^−7^ M alone and in combination with miR-375 inhibitor. Moreover, the ability of OCT to induce cell apoptosis alone and in combination with miR-375 inhibitor was explored by flow cytometry. Finally, the intracellular signaling pathways involved in cell proliferation (ERK1/2) and apoptosis (Caspase 3 and PARP) were evaluated by WB in AtT20/D16.

### Compounds

OCT was supplied by Selleckchem (code number SM201-995). PAS was obtained from Recordati Rare Diseases. DEX, a potent GC, was provided by Sigma Aldrich (code number D2915). All drugs were dissolved in sterile phosphate-buffered saline (PBS) 1× (SIAL, code number SIAL-PBS-1A) at a concentration of 10^−3^ M and stored at −20 °C. Fresh aliquots were thawed for each experiment, and serial dilutions were prepared in Dulbecco’s modified Eagle’s medium (DMEM) full (Gibco, code number 11965092) for each new experiment.

### Tissue and Serum Samples

At the endocrinology center of the University of Naples “Federico II”, patients with active CD were considered eligible and enrolled in the study. Blood samples from 26 patients diagnosed with active CD and 17 age- and sex-matched healthy subjects (Table 1) were used for the analysis of circulating miR-375. All participants enrolled in the study provided signed informed consent (EC Prot. 359/18). Six human corticotroph pituitary tumors and 2 normal pituitary tissues (Table 2) were included in the study for analysis of tissue miR-375. The 2 normal pituitaries obtained postmortem from 2 healthy donors (healthy White men aged 42 and 61 years old with no endocrine or metabolic diseases, 1 death for gastrointestinal bleeding and the other for brain edema) were supplied by the Global Biobank and Human Biospecimen CRO Cureline (Cureline, San Francisco, USA).

**Table 1.** Clinical features of patients with CD whose serum samples were used for circulating miR-375 analysis

	Healthy subjects	CD patients
n	17	26
Sex F (%) : M (%)	11 (64.70%) : 6 (35.30%)	18 (69.23%) : 8 (30.77%)
Age mean ± SEM	44.88 **±** 2.90	44.96 ± 2.67
BMI mean ± SEM	24.26 ± 0.87	65.81 ± 22.6***
ACTH pg/mL (09:00 hours) mean ± SEM	NA	156 ± 50.83
Cortisol ng/mL (09:00 hours) mean ± SEM	NA	204.9 ± 25.59
24 hours UFC (ULN mean)	NA	2.48 ± 0.37

Abbreviations: ACTH, adrenocorticotroph hormone; BMI, body mass index; CD, Cushing disease; NA, not available; UFC, urinary free cortisol; ULN, upper limit of normal.

***P = .0004 vs healthy subjects.

**Table 2.** Clinical features of patients with CD whose pituitary tumors were used for tissue miR-375 analysis

n	6
Sex F(%) : M(%)	5 (83.3%): 1 (16.7%)
Age mean ± SEM	39.5 ± 3.4
ACTH pg/mL (09:00 hours) mean ± SEM	56.3 ± 6.0
Cortisol ng/mL (09:00 hours) mean ± SEM	47.4 ± 23.6
24 hours UFC (ULN mean)	2.5 ± 0.83

Abbreviations: ACTH, adrenocorticotroph hormone; CD, Cushing disease; UFC, urinary free cortisol; ULN, upper limit of normal.

### Cell Lines and Primary Pituitary Cultures

AtT20/D16 cell line (CRL-1795), a strain of the mouse corticotroph pituitary tumor model AtT20, and GH3 cell line (CCL-82.1), a rat somatotroph pituitary tumor model, were provided by American Type Culture Collection (ATCC) and cultured in DMEM media supplemented with 10% fetal bovine serum (FBS) (Gibco, code number 16140071), 200 mM L-glutamine (Gibco, code number 25030081) and 1 × 10^5^ U/L penicillin and streptomycin (Gibco, code number 15140122). Both cell lines were tested for mycoplasma and grown in a humidified condition at 37 °C and 5% CO_2_.

Two human pituitary primary cultures were obtained by transsphenoidal surgery from a female and a male patient with CD due to a corticotroph microadenoma operated at the Neurosurgical Center of the Fondazione Istituto di Ricovero e Cura a Carattere Scientifico (IRCCS) Ca’ Granda Ospedale Maggiore Policlinico of Milan. ACTH-dependent hypercortisolism was biochemically established according to guidelines (15) based on at least 2 of the following abnormal tests: high 24-hour urinary free cortisol (UFC) levels, loss of circadian rhythm in salivary cortisol, and lack of cortisol suppression after 1 mg of DEX overnight, in association with detectable baseline ACTH plasma levels (>20 ng/L). Differential diagnosis of ACTH-dependent hypercortisolism was established through (1) the corticotropin-releasing hormone (CRH) test (positive response: ACTH and/or cortisol plasma levels increase by more than 50% and/or 20%, respectively); and (2) the high-dose DEX suppression test (positive response: serum cortisol levels reduction to a value of <50% of the basal level (Table 3).

**Table 3.** Clinical features of patients with CD from whom primary cultures of pituitary tumors were obtained

Patient no.	Sex, age	ACTH pg/mL	24 hours UFC (ULN)
1	F, 47	44.9	2.1
2	M, 15	35.3	6.9

Abbreviations: ACTH, adrenocorticotroph hormone; CD, Cushing disease; UFC, urinary free cortisol; ULN, upper limit of normal.

Tissues were enzymatically dissociated in DMEM containing 2 mg/mL collagenase (Sigma Aldrich, code number C9891-500MG) at 37 °C for 2 hours, as previously described (16). Dispersed cells were cultured in DMEM supplemented with 10% FBS, 2 mM glutamine, and 1 × 10^5^ U/L penicillin and streptomycin.

### miR-375 Isolation From CD Patients, Corticotroph Pituitary Tissues, and Cell Lines

#### miR-375 isolation from patients’ serum

Twenty-six patients with active CD and 17 age- and sex-matched healthy subjects were enrolled in the study. Patients and controls provided morning serum samples that were centrifuged at 4 °С and 2000 rpm for 5 minutes within 2 hours of collection to preserve miRNA integrity, then aliquoted and stored at −80 °C until further analysis. Circulating miRNAs were isolated from 200 µL of human serum using miRNeasy Serum/Plasma Advanced Kit (Qiagen, code number 217204) as previously described (17). Recovered miRNAs (50 ng/µL) were used for cDNA synthesis using the miScript system (Qiagen, code number 218161), followed by cDNA preamplification. cDNA derived from the preamplification reaction was used as a template for RT-qPCR analysis to evaluate circulating human miR-375 (Qiagen, hs_miR-375_2, MS00031829) expression levels using miScript SYBR Green kit (Qiagen, code number 218075). RT-qPCR was performed as described above. Circulating relative miR-375 expression levels in each sample were normalized using the spike-in control Cel-miR-39-3p.

#### miR-375 isolation from corticotroph pituitary tissues

Tissue miR-375 expression levels were also evaluated in 6 human corticotroph pituitary tumors and 2 normal pituitaries. A mortar and pestle, previously sterilized, were used to pulverize about 0.3 mg of each tissue sample in liquid nitrogen; samples were crushed until a fine powder was obtained. The powder obtained was placed into a new cold microtube and miRNAs were isolated using miRNeasy Mini Kit (Qiagen, code number 217004). High-quality miRNAs were then eluted in 20 µL of RNase-free water. miRNAs (250 ng/µL) isolated from cell cultures were reverse transcribed using miScript II RT Kit (Qiagen, code number 218161). The miScript Reverse Transcriptase mix (20 µL final volume) contains an internal synthetic RNA control (miRNA reverse transcription control, miRTC) to assess reverse transcription performance during RT-qPCR. cDNA derived from the reverse-transcription reaction was used as a template for RT-qPCR analysis to evaluate miR-375 expression using a human-specific miScript primer assay to detect hs_miR-375_2 (Qiagen, MS00031829) and the housekeeping U6 snRNA (*RNU6-1*) (Qiagen, Hs_RNU6_11, MS00033740). The PCR mix was prepared by adding 6.5 µL 2× QuantiTect SYBR Green PCR Master mix, 1.25 µL of universal primer, 1.25 µL of miScript primer assay, 2 µL of cDNA, and 1.5 µL of H_2_O. Then, 12.5 µL of PCR mix was distributed to each well to charge 2.5 ng of cDNA per well. The StepOne Plus Real-Time PCR system instrument was used to run the RT-qPCR applying the following protocol: initial activation step at 95 °C (15 minutes), then samples were subjected to 40 cycles of denaturation at 94 °C (15 seconds), annealing at 55 °C (30 seconds) and extension at 70 °C (30 seconds). Results were expressed as the mean of 3 independent experiments.

#### miR-375 isolation from cell cultures

miRNAs were isolated from AtT20/D16 and GH3 cell lines as previously described (17). Briefly, cells were plated into 60-mm culture dishes in complete medium. After 24 hours of adhesion, miRNAs were isolated using miRNeasy Mini Kit (code number 217004; Qiagen, Italy). Both cell lines were evaluated in basal conditions; moreover, miR-375 expression was also investigated in AtT20/D16 treated with DEX 10^−8^ M for 24 hours, 72 hours, and 6 days. miR-375 expression was evaluated using a mouse-specific miScript primer assay to detect Mm_miR-375_2 (Qiagen, MS00032774) and the housekeeping U6 snRNA (*Rnu6-1*) (Qiagen, Mm_RNU6_11, MS00033740) was used to normalize miR-375 expression.

### Bioinformatic Analysis of miR-375 Target Genes

Sequence analysis was performed to evaluate a potential seed sequence that matches human *SSTR2* mRNA and Sstr2 mouse mRNA. Human *SSTR2* and mouse *Sstr2* and miR-375 sequences were downloaded from the Ensembl database (ENST00000357585.4 and ENSMUST00000067591.3) and miRbase database (MI0000783 and MI0000792), respectively.

### Cell Line Transfections and Drug Treatments

AtT20/D16 and corticotroph pituitary tumor cells of 2 human primary cultures were transfected by using HilyMax reagent reconstituted by Lipoform Buffer Dojindo, (Dojindo Laboratories, code #357), and miScript miRNA inhibitor at the final concentration of 75 nM (Qiagen, miRCURY LNA miRNA inhibitor hsa-miR-375) reconstituted with 100 μL of TE buffer 1×, and miScript miRNA mimic at the final concentration of 100 nM (Qiagen, miRCURY LNA miRNA mimic hsa-miR-375) reconstituted with 75 μL of nuclease-free sterile water, following the supplier's instructions.

After 24 hours of adhesion, AtT20/D16 cells were starved for 2 hours by replacing the full medium with DMEM serum-free medium. After starvation, AtT20/D16 cells were transfected with a final concentration of miR-375 inhibitor or mimic at concentrations of 75 and 100 nM, respectively, for 24 hours in complete medium. Drug treatments were performed after 24 hours of transfection as described in the following sections.

### mRNA Isolation and RT-qPCR

Cells were plated in 60-mm culture dishes in a complete culture medium and incubated at 37 °C in a humidified 5% CO_2_ atmosphere. After 24 hours of adhesion, total RNA was extracted and isolated from cell lines with the use of Dynabeads Oligo (dT)25 (Dynal AS), as previously described (18). RT-qPCR was performed to quantify the basal *Sstr2* mRNA expression levels in both AtT20/D16 and GH3 cell lines and of DEX-dependent *Sstr2 m*RNA expression in AtT20/D16 cell line. Primer sequences are listed in Table 4. The total reaction volume (12 μL) consisted of 5 μL of cDNA, 0.5 μL of primers (1 μM) and 6.5 μL 1× Sybr Green Mix (Thermo Fisher Scientific Inc., Maxima SYBR Green qPCR Master Mixes). RT-qPCR was performed in 96-well optical plates on a StepOne Plus real-time PCR machine (Applied Biosystems) according to the following protocol: after 2 initial heating steps at 50 °C (2 minutes) and 95 °C (10 minutes), samples were subjected to 40 cycles of denaturation at 95 °C (15 seconds) and annealing at 60 °C (60 seconds). All samples were assayed in duplicate. Values were normalized against the expression of the housekeeping genes β-actin (*Actb*) or cyclophilin A (*CypA*). To exclude genomic DNA contamination in RNA extracts, cDNA reactions were also performed without reverse transcriptase and amplified with each primer pair. Results were expressed as a mean ± SEM of 3 independent experiments.

**Table 4.** Primers sequences

Gene	Primers
Mm_*Sstr2*	Fw: GGCGTGGTACACAGGTTT Rv: GAAGACAGCCACTACGATGG
Rn_*Sstr2*	Fw: GGTTATCCTCACCTACGCCA Rv: GTCCTGCTTACTGTCGCTCC
Rn/Mm_*Actb*	Fw: ACAGCTTCACCACCACAGCTGA Rv: GAGGTCTTTACGGATGTCAACGTC
Mm_*CypA*	Fw: CGCCACTGTCGCTTTTCG Rv: AACTTTGTCTGCAAACAGCTC

Abbreviations: Mm, Mus musculus; Rn, Rattus norvegicus.

### Protein Extraction and Western Blot

Protein extraction and WB were performed, as previously described (18). Briefly, AtT20/D16 cells were seeded into 100 mm culture dishes at 2 × 10^6^ cell density in complete medium. After 24 hours of adhesion, cells were starved for 2 hours and transfected for 24 hours. Protein extraction and blot for SSTR2 expression were evaluated after 24 hours of transfection, whereas pERK1/2, ERK, caspase 3, and PARP were evaluated after 3 days of drug treatment. Proteins (20 μg) were loaded, respectively, into polyacrylamide gel. Primary antibodies used for WB analysis were the following: SSTR2 (Santa Cruz Biotechnology Cat# sc-365502, RRID:AB_10859216 dilution used 1:500); pp44/42 ERK1/2 (Thr202/Tyr204) (Santa Cruz Biotechnology Cat# sc-7383, RRID:AB_627545, dilution used 1/500), p44 ERK2 (Santa Cruz Biotechnology Cat# sc-1647, RRID:AB_627547, dilution used 1/500); Caspase3 (Cell Signaling Technology Cat# 14220, RRID:AB_2798429, dilution used 1:1000); PARP (Cell Signaling Technology Cat# 9542, RRID:AB_2160739, dilution used 1/1000), and ACTB (Sigma-Aldrich Cat# A4700, RRID:AB_476730, dilution used 1/10000); whereas antimouse and antirabbit horseradish peroxidase–conjugated secondary antibodies (ImmunoReagents, code number GtxRB-003-DHRPY) dilution used 1:2000 in 2.5% nonfat dry milk in PBS 1× for the detection of proteins. Primary antibodies were probed on nitrocellulose filters overnight, while peroxidase-conjugated secondary antibodies were probed on nitrocellulose filters for 2 hours. Immunoreactive bands after chemiluminescent reaction by an ECL system (Millipore, Immobilon Western, code number WBKLS0500) were detected using ImageQuant Las 4000 (GE Healthcare). The data in the graphs are expressed as a percentage of control and represent the mean ± SEM of 8 independent experiments.

### Immunofluorescence Staining

AtT20/D16 cells were seeded in 35-mm dishes (Ibidì, code number 80136) at 5 × 10^4^ cell density. After 24 hours of adhesion, cells were starved for 2 hours, transfected for 24 hours with miR-375 inhibitor and mimic, and treated with OCT 10^−7^ M for 20 minutes. After treatment, cells were washed twice with PBS 1×, and then fixed in 4% paraformaldehyde for 30 minutes at 4 °C. Subsequently, cells were washed thrice in PBS 1× and incubated in permeabilization buffer (Triton 0.25%/PBS 1×) for 10 minutes. After subsequent thrice washing in PBS 1×, slides were placed in a blocking buffer (15% FBS/PBS 1× and 0.1%/Triton/PBS 1×) for 1 hour at RT. Subsequently, AtT20/D16 cells were incubated with primary antibodies against SSTR2 (Abcam Cat# ab134152, RRID:AB_2737601, dilution used 1:200) for 2 hours at room temperature. Then cells were washed thrice in 0.1% Triton/PBS 1× and incubated 1 hour with TRITC-conjugated secondary antibodies (ImmunoReagents, code number GtxRb-003-DRHOX, diluted 1:500) in blocking buffer. To detect the nuclei, a 4.6-diamidino-2-phenylindole (DAPI) (Sigma Aldrich, code number MBD0015) staining, diluted in PBS 1× 1:60000, was used. Images were visualized on an inverted microscope Olympus IX51 equipped for fluorescence and phase-contrast microscopy (Olympus) and were captured at 60× magnification and acquired with Olympus Digital Camera F-View II (Evident Scientific).

Primary cultured cells were seeded on poly-L-lysine–coated coverslips in complete medium. Due to the limited cell number, SSTR2 protein expression has been evaluated in primary cultured cells in basal conditions and after treatment with miR-375 inhibitor. After treatment with miR-375 inhibitor, primary cultured cells were washed thrice with PBS 1×, fixed in 4% paraformaldehyde for 10 minutes at 4 °C, and incubated with blocking buffer (5% FBS, 0.3% Triton X-100, in PBS) for 1 hour at room temperature. Cells were then overnight incubated with anti-SSTR2 (Abcam Cat# ab134152, RRID:AB_2737601, diluted 1:50) primary antibody at 4 °C. After that, cells were washed 3 times with PBS 1× and incubated with antirabbit Alexa Fluor 488-conjugated secondary antibody (Thermo Fisher Scientific, code number A11008, RRID:AB_143165, diluted 1:500) at room temperature for 2 hours. Both primary and secondary antibodies were diluted in Antibody Diluent Reagent Solution (Thermo Fisher, code number 003118). Coverslips were mounted on glass slides using a mounting medium containing DAPI. Images were captured by a Confocal Laser Scanning Microscope FV4000 (Evident Scientific) at a 60× magnification, and IF analysis was performed with ImageJ software, according to the following protocol (https://theolb.readthedocs.io/en/latest/imaging/measuring-cell-fluorescence-using-imagej.html). The data presented in the graphs are expressed as a percentage of fluorescence intensity normalized to the cell number. Data represent the mean ± SEM of 3 independent experiments.

### Cell Proliferation Assay

DNA assay was performed as previously described (19). Briefly, AtT20/D16 cells were seeded in 24 well plates at a density of 2 × 10^5^ in DMEM full. After 24 hours of adhesion, AtT20/D16 cells were starved for 2 hours, transfected for 24 hours, and treated with OCT and PAS at concentrations 10^−8^ M and 10^−7^ M alone and in combination with miR-375 inhibitor. After 3 days of treatment, measurement of total DNA content, representative of the number of cells, was performed using the bisbenzimide fluorescent dye (Hoechst 33258) (Boehring Diagnostics), as previously described (19). Six replicated wells were performed for each analysis, and at least 3 independent experiments were performed.

### Flow Cytometry Analysis of Apoptosis

AtT20/D16 cells were seeded into 60-mm culture dishes in complete medium. After 24 hours of adhesion, AtT20/D16 cells were treated as described above. After 3 days of treatment, apoptosis analyses were performed using the FITC Annexin V Apoptosis Detection kit (BD Bioscience, code number 556547) according to the manufacturer's instructions. Stained cells were analyzed using BD Accuri C6 plus flow cytometry. The data presented in the graphs are expressed as a percentage and represent the mean ± SEM of 3 independent experiments.

### Statistical Analysis

All statistical analyses were performed using GraphPad software. The results are expressed as the mean ± SEM. An unpaired 2-tailed nonparametric Student t-test was used to assess the significance between 2 series of data, followed by the Mann–Whitney test to compare ranks. *P* < .05 was considered statistically significant.

## Results

### Circulating, Tissue, and Cellular miR-375 Expression

**Figure 1.**
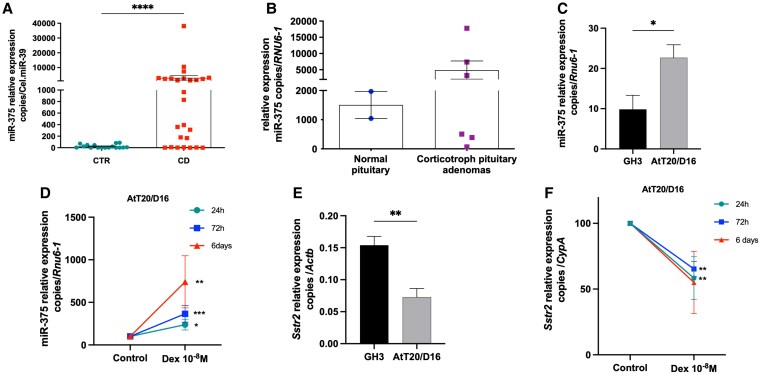
(A) Expression of circulating miR-375 in sera of 17 normal subjects and 26 patients with CD. (B) Expression of miR-375 in 2 normal pituitaries and 6 corticotroph pituitary adenomas tissues. (C) Expression of miR-375 in GH3 and AtT20/D16 cell lines. (D) Expression of miR-375 in AtT20/D16 cell line under DEX 10^−8^ M treatment after 24 hours, 48 hours and 6 days. (E) *Sstr2* gene expression in GH3 and AtT20/D16 cell lines. (F) *Sstr2* gene expression modulation by DEX 10^−8^ M treatment after 24 hours, 48 hours, and 6 days in AtT20/D16 cell line. Data in the graphs performed in cell lines represent mean ± SEM of 3 independent experiments. *P < .05, **P < .01, ***P < .001, ****P < .0001 vs control or among the groups.

As shown in Fig. 1, circulating expression evaluation revealed significantly higher miR-375 levels (*P* < .0001) in patients with CD compared with healthy subjects (Fig. 1A), whereas miR-375 tissue levels appeared to be not significantly higher in human corticotroph pituitary tumors compared with normal pituitary, probably due to the limited number of samples or to the cellular heterogeneity in normal pituitary tissue, compared with corticotroph tumor tissues, potentially influencing the measurement of miR-375 expression (Fig. 1B). Interestingly, miR-375 expression levels in the AtT20/D16 cell line were significantly higher than those observed in the GH3 cell line (*P* < .05) (Fig. 1C). In AtT20/D16, treatment with DEX 10^−8^ M significantly increased miR-375 expression after 24 hours (+138.7%, *P* = .02), 72 hours (+265.3%, *P* = .0002), and 6 days (+641.1%, *P* = .002) of treatment (Fig. 1D). These findings demonstrated that under the condition of GC excess, both circulating and tissue miR-375 levels can be significantly increased, as established by the significantly higher serum miR-375 levels in patients with CD, as well as by the significantly higher tissue miR-375 levels in murine corticotroph pituitary tumor cell model.

### *Sstr2* mRNA Expression in AtT20/D16 and GH3 Cell Lines

As shown in Fig. 1, *Sstr2* mRNA evaluation revealed significantly lower levels in AtT20/D16 than in the GH3 cell line (*P* = .005) (Fig. 1E). Furthermore, the treatment of the AtT20/D16 cell line with DEX 10^−8^ M induced a significant reduction in *Sstr2* mRNA expression after 24 hours (41.8%, *P* < .008) and 72 hours (34.7%, *P* < .002), whereas no statistically significant reduction in the *Sstr2* mRNA expression was observed at 6 days of treatment (Fig. 1F). These findings corroborate the hypothesis that GC excess might downregulate *Sstr2* expression. However, the extent to which this effect depends on indirect GC action via epigenetic mechanisms involving miRNAs has not been demonstrated yet.

### miR-375 Regulates SSTR2 Protein Expression in AtT20/D16 and in Human Corticotroph Pituitary Tumor

**Figure 2.**
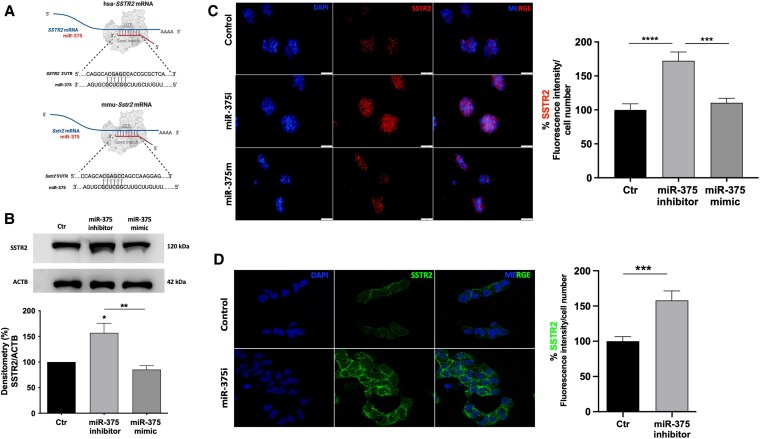
(A) Scheme of seed match between miR-375 and *Sstr2* in mouse and *SSTR2* in human. (B) Protein expression and densitometry of SSTR2 in AtT20/D16 treated with miR-375 inhibitor and mimic, evaluated by WB. The data in the graphs are expressed as a percentage of control and represent the mean ± SEM of 8 independent experiments. (C) Protein expression and fluorescence intensity of SSTR2 in AtT20/D16 treated with miR-375 inhibitor and mimic and in 2 human corticotroph primary cultures and (D) treated with miR-375 inhibitor evaluated by IF. The data in the graphs are expressed as a percentage of control normalized to cell number and represent the mean ± SEM of 3 independent experiments in cell lines. *P < .05, **P < .01, ***P < .001, ****P < .0001 vs control or among the groups.

To verify whether SSTR2 is a potential target gene of miR-375, miRTarBase, TargetScan and miRPath Diana Tools were used. However, these tools predict a base pairing pattern only between miRNA and the 3′ untranslated region of the target mRNA sequence. For this reason, SSTR2 is not reported as a target gene of miR-375, neither in humans nor in mice. Nevertheless, the analysis of the complementarity between miR-375 and *SSTR2* in humans and *Sstr2* in mice (by Ensembl database [ENST00000357585.4 and ENSMUST00000067591.3] and miRbase database [MI0000783 and MI0000792], respectively) shows that several potential noncanonical miR-mRNA binding sites could therefore regulate the genetic and protein expression of SSTR2 (Fig. 2A).

As shown in Fig. 2, WB and IF revealed that inhibition of miR-375 induced a significant increase (+57%, *P* = .031 by WB; + 72.2%, *P* = .0001 by IF) compared with control whereas miR-375 mimic transfection did not induce any changes (−14.7% by WB; + 10.5% by IF) in SSTR2 protein expression in AtT20/D16 cell line (Fig. 2B and 2C). Accordingly, IF revealed that inhibition of miR-375 induced a significant increase (+57.9%, *P* = .0007) in SSTR2 protein expression in cells from human corticotroph pituitary tumor (Fig. 2D). These results demonstrated that miR-375 regulation may control SSTR2 protein expression in both human and mouse corticotroph cell models.

### Effects of Octreotide and Pasireotide on Parental and Transfected AtT20/D16 Cell Line

To assess the function of the miR-375-dependent SSTR2 higher expression levels, AtT20/D16 were treated with OCT and PAS at concentrations of 10^−8^ M and 10^−7^ M, alone or in combination with miR-375 inhibitor, for 3 days.

**Figure 3.**
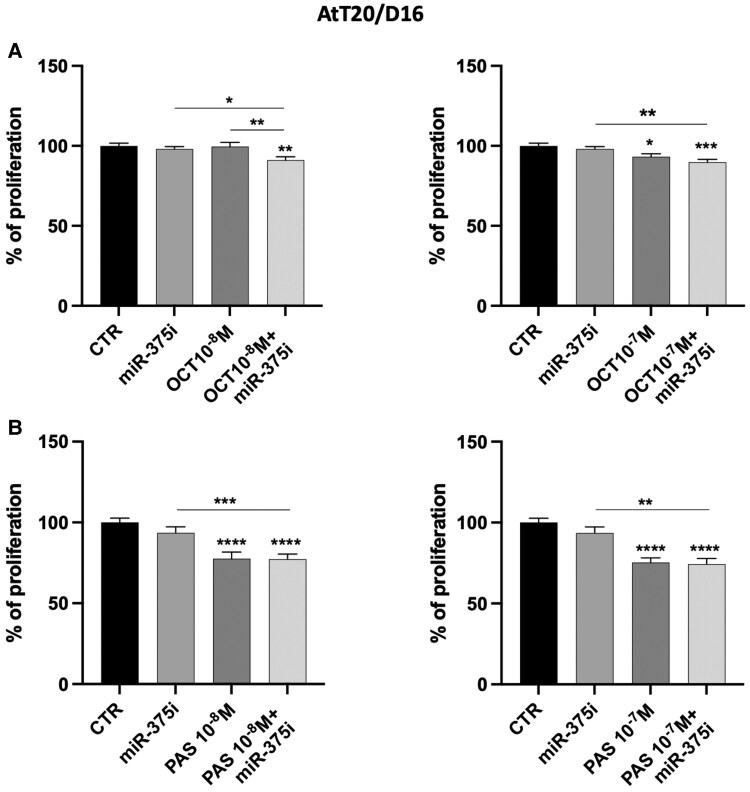
Evaluation of cell proliferation in AtT20/D16 treated with (A) OCT 10^−8^ M and 10^−7^ M and (B) PAS 10^−8^ M and 10^−7^ M with and without miR-375 inhibitors after 3 days of treatment by DNA assay. The data in the graphs are expressed as a percentage of control and represent the mean ± SEM of 3 independent experiments. *P < .05, **P < .01, ***P < .001, ****P < .0001 among the groups.

As shown in Fig. 3, OCT 10^−8^ M did not reduce cell proliferation, whereas OCT 10^−7^ M demonstrated a slightly significant reduction (6.8%, *P* < .05) in AtT20/D16 cell proliferation compared with untreated cells (Fig. 3A). Interestingly, when AtT20/D16 cells were cotreated with OCT 10^−8^ M or 10^−7^ M and miR-375 inhibitor, both OCT concentrations were able to significantly reduce AtT20/D16 cell proliferation (OCT 10^−8^ M + miR-375 inhibitor 9.0%, *P* < .0023 vs control, *P* < .0058 vs OCT 10^−8^ M and *P* < .025 vs miR-375 inhibitor; OCT 10^−7^ M + miR-375 inhibitor 10.1%, *P* < .0001 vs control, *P* < .0025 vs miR-375 inhibitor). These results demonstrated that the inhibition of miR-375 affects OCT sensitivity in AtT20/D16 cells.

As expected for its preferential SSTR5 binding, PAS alone at concentrations of 10^−8^ M and 10^−7^ M significantly reduced (22.7% and 24.7%, *P* < .0001, respectively) AtT20/D16 cell proliferation compared with untreated cells (Fig. 3B). Nevertheless, when AtT20/D16 cells were cotreated with PAS 10^−8^ M or 10^−7^ M and miR-375 inhibitor, no additive effect was observed. These results demonstrated that the inhibition of miR-375 does not affect PAS sensitivity in AtT20/D16 cells.

### miR-375 Inhibition Affects SSTR2 Protein Expression and Cell Localization in Basal and Treated AtT20/D16

**Figure 4.**
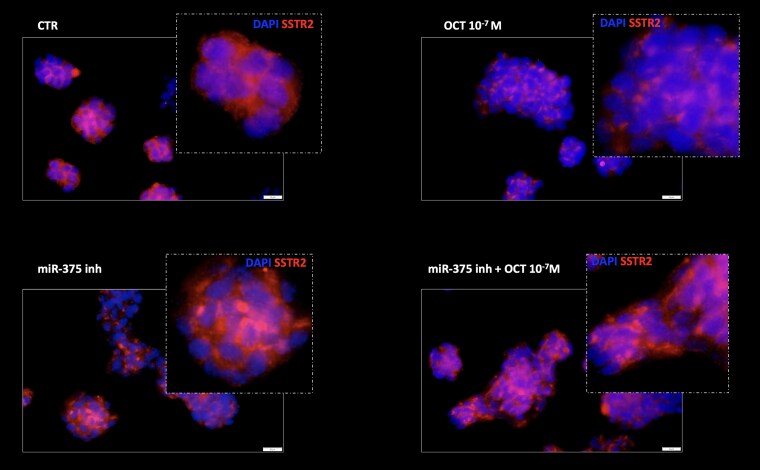
Cellular localization of SSTR2 protein expression in AtT20/D16 evaluated by IF in the presence of OCT 10^−7^ M for 20 minutes, miR-375 inhibitor and their combination. Images were captured at 60× magnification. The images reported in the figure represent 1 of the 3 independent experiments.

To investigate the effect of miR-375 on SSTR2 expression and localization, and thus on OCT ability to inhibit cell proliferation, AtT20/D16 cells were transfected with miR-375 inhibitor for 24 hours and then treated with OCT 10^−7^ M for 20 minutes, alone and in combination, and IF was established. As shown in Fig. 4, at basal condition, AtT20/D16 cells expressed SSTR2 protein mainly at the membranous level, but when treated with OCT 10^−7^ M, AtT20/D16 cells mainly displayed punctate cytoplasmic staining of SSTR2 protein. As expected, when AtT20/D16 cells were treated with miR-375 inhibitor SSTR2 protein expression was higher and mainly localized on the membrane. Interestingly, when AtT20/D16 cells were cotreated with miR-375 inhibitor and OCT 10^−7^ M, the intensity of the fluorescence demonstrated a higher expression of SSTR2 mainly localized at the cytoplasmic level, suggesting that the increased SSTR2 levels induced by the miR-375 inhibitor positively affect the antiproliferative properties of OCT induced by receptor activation and internalization.

### miR-375 Inhibition Affects the Sensitivity to Octreotide by Modulating Cytostatic and Apoptotic Signaling Pathways in AtT20/D16

As OCT activates apoptotic and cytostatic actions involving several signal transduction effectors including caspase 3 and PARP and MAPK, respectively, early and late apoptosis was evaluated by flow cytometry analysis and Caspase 3 and PARP cleavage, together with ERK1/2 phosphorylation were investigated in AtT20/D16 cells treated with miR-375 inhibitor, OCT 10^−8^ M and OCT 10^−7^ M alone and in combination.

**Figure 5.**
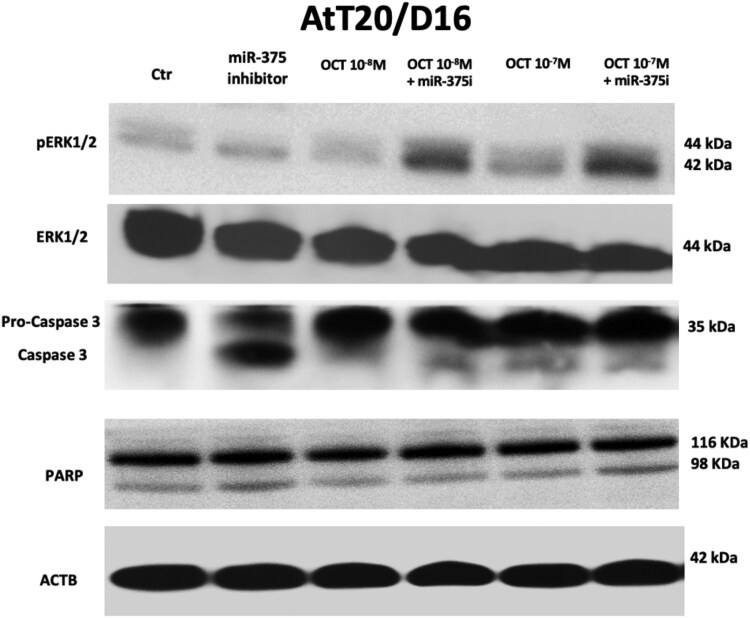
Intracellular signaling modulation of proliferation markers, ERK1/2, and apoptotic markers, caspase 3, and PARP, in AtT20/D16 treated with OCT 10^−8^ M and 10^−7^ M, miR-375 inhibitor, and their combination, evaluated by WB.

Flow cytometry analyses with annexin V demonstrated that the treatment with OCT 10^−8^ M and OCT 10^−7^ M alone increased AtT20/D16 percentage in early and late apoptosis compared with untreated cells. Interestingly, while the cotreatment of miR-375 inhibitor and OCT 10^−8^ M increased only the percentage of cells in late apoptosis compared with the OCT 10^−8^ M single treatment, the combination of miR-375 inhibitor and OCT 10^−7^ M boosted both percentages of cells in early and late apoptosis compared with the OCT 10^−7^ M single treatment, accordingly with the cell proliferation inhibition (20). Moreover, as shown in Fig. 5 the apoptotic effect observed with OCT 10^−7^ M in the presence of miR-375 inhibitor was accompanied by induction of Caspase 3 and PARP cleavage. Interestingly, cotreatment of miR-375 inhibitor with both OCT 10^−8^ M and OCT 10^−7^ M enhanced the phosphorylation of ERK1/2, potentiating the cytostatic effect of OCT (Fig. 5).

## Discussion

**Figure 6.**
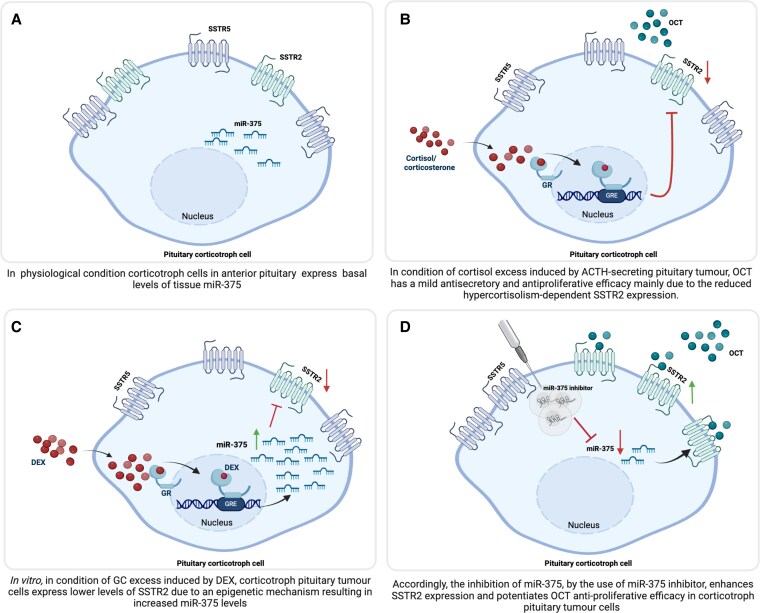
A graphical cartoon (A-D) summarizes the background, rationale and goal of the current study. OCT, octreotide; DEX, dexamethasone; GR, glucocorticoid receptor.

The findings of the current study confirm the expression of miR-375 in the AtT20/D16 murine corticotroph pituitary tumor model (Fig. 6A) and support the hypothesis that GC excess regulates SSTR2 expression in AtT20/D16 and human corticotroph pituitary primary culture via epigenetic mechanisms involving miR-375. Moreover, the results of the present study demonstrate that the negative modulation of miR-375, by positively impacting on SSTR2 expression in human and murine corticotroph pituitary tumor cell models, potentiates the OCT antiproliferative effect (Fig. 6B-6D).

*SSTR5* is the predominant receptor subtype detected in human corticotroph pituitary tumors, which display minor expression of *SSTR2* (21). Conversely, human somatotroph pituitary tumors predominantly express *SSTR2* subtype with higher levels than *SSTR5* (3, 5, 22-30). Accordingly, in the current study, the comparison of the most utilized murine corticotroph and somatotroph pituitary tumor cell lines, AtT20 and GH3, respectively, demonstrated that the murine corticotroph cell line AtT20 expresses lower levels of *Sstr2* than those observed in the murine somatotroph pituitary tumor cell line GH3. In line with these data, the second-generation SRL PAS is the only pituitary-targeted drug approved for the treatment of CD in which the consequential ACTH-dependent hypercortisolism is hypothesized to downregulate pituitary tumor SSTR2 expression, limiting the efficacy of first-generation SRL treatment in patients with CD (22). The hypothesis that SSTR2 is downregulated by cortisol excess is supported by several in vitro and ex vivo observations. In human corticotroph pituitary tumor organoids, generated by induced human pluripotent stem cells, Mallick et al reported that DEX relevantly reduce *SSTR2* expression and, surprisingly, also *SSTR5* expression (31). On the other hand, the nonselective GC receptor antagonist mifepristone and the selective GC receptor modulator relacorilant were reported to significantly stimulate the expression of *SSTR2* and *SSTR5* receptors via noncanonical sonic hedgehog signaling involving GLI1, and precisely, mifepristone led to a greater induction of *SSTR2* than relacorilant, whereas relacorilant led to a greater induction of *SSTR5* than mifepristone (31). Moreover, DEX inhibited pituitary *Sstr2* expression in a murine model, both in vivo and in vitro (32, 33). Furthermore, in AtT20 cell model, relacorilant not only restored the DEX-mediated reduction of *Sstr2* but further induced *Sstr2* overexpression in a concentration-dependent manner (9). In contrast, mifepristone restored the GC-mediated *SSTR2* expression reduction without further positively affecting *SSTR2* expression in human neuroendocrine cell lines (4). Despite variable findings, these data demonstrated a direct role of GCs, and their local modulation, on pituitary corticotroph cells *SSTRs* expression. Interestingly, in humans, the comparison of *SSTR2* and *SSTR5* expression performed in pituitary tumor samples from patients with CD with hypercortisolism and from patients with normalized preoperative cortisol levels demonstrated that the *SSTR2* mRNA, but surpisingly not the protein, was significantly higher in the latter group than in the samples of patients with hypercortisolism before surgery (30), suggesting that the endogenous systemic cortisol levels may modulate at least the *SSTR2* transcription at pituitary level. In agreement with the literature, the results of the current study established that DEX treatment reduces *Sstr2* expression in AtT20/D16 cell line, confirming the hypothesis that *Sstr2* is downregulated by GCs (33), as demonstrated also in different neuroendocrine tumor models, such as TT and BON human cell lines (4).

Nevertheless, the molecular mechanism underlying the regulation of SSTR2 by GC excess remains largely unelucidated. The current study provides evidence of the epigenetic mechanism involvement in GC-dependent SSTR2 regulation in human and murine corticotroph cell models, specifically by controlling miRNA expression. Previous data (14) and the results of the current study demonstrated that DEX treatment increased levels of miR-375 in AtT20 cells. Bioinformatic analysis revealed noncanonical seed sequences for human *SSTR2* and mouse *Sstr2* in miR-375, suggesting a potential regulation of SSTR2 protein expression and function by miR-375. This hypothesis was also strengthened by the observation of lower *Sstr2* expression in AtT20 cells than in GH3 cells, of increased circulating levels of miR-375 in patients with CD compared with healthy subjects, and of an increasing trend of tissue miR-375 expression in corticotroph pituitary tumors compared with normal pituitaries.

In this context, the present study provides evidence of the involvement of miR-375 in the SSTR2 expression, demonstrating for the first time that the inhibition of miR-375 is sufficient to increase SSTR2 expression in AtT20/D16 cell model and in human corticotroph pituitary tumor cells from 2 primary cultures.

SRLs are largely used in clinical practice for the inhibition of hormone secretion, cell proliferation and tumor growth in a range of pituitary tumors. However, the different pituitary tumor histotypes heterogeneously coexpressed the different SSTRs subtypes with a consequent variance in SRL responsiveness. As discussed above, patients with untreated CD are by definition characterized by hypercortisolism that causes low *SSTR2* expression levels in the pituitary tumor tissue, negatively affecting the response to OCT treatment. Indeed, in AtT20 cells, pretreatment with DEX lowered the potency of OCT to reduce ACTH secretion and the maximum binding capacity of the radioligand [125I-Tyr3]OCT to SSTR2 (33). These observations may, at least in part, explain why OCT is generally ineffective in patients with untreated CD (3, 34-36) and let the researcher hypothesize that control of cortisol production induced by steroidogenesis inhibitors could reverse SSTR2 expression and increase the efficacy of the tumor-directed therapy with OCT. In humans, the normalization of endogenous cortisol levels achieved by treatment with steroidogenesis inhibitors, such as ketoconazole, before undergoing pituitary surgery, did not further increase the in vitro antisecretory efficacy of first-generation SRLs (30); moreover, ketoconazole demonstrated no direct effect on *SSTRs* modulation on induced human pluripotent stem cells (31). These data suggested that neither ketoconazole direct action on pituitary corticotroph cells nor the systemic normalization of endogenous cortisol levels induced by ketoconazole in patients with CD was sufficient to potentiate the efficacy of first-generation SRLs. To better verify this hypothesis, a proof of concept study was performed on patients with CD adopting a sequential treatment strategy with ketoconazole, to reduce cortisol levels, followed by OCT as maintenance therapy once cortisol levels were normalized (37). The study results demonstrated that OCT effectively sustained normal urinary cortisol levels in 27% of patients (responders), while 36% of patients showed a 50% reduction in urinary cortisol levels and were defined partial responders to OCT, and only 4 patients developed hypercortisolism after ketoconazole discontinuation (nonresponders), demonstrating that OCT effectively maintains normal cortisol production, although in a subset of patients with mild CD (37). Therefore, the effect of treatment with steroidogenesis inhibitors on the regulation of SSTRs expression is still doubtful, and the greater efficacy of the use of the firt-generation SRLs, administered after steroidogenesis inhibitors, which has been observed only in a subgroup of CD patients with mild hypercortisolism, requires further investigation. Conversely, in line with the predominant expression of *SSTR5* in human corticotroph pituitary tumors and the negligible impact of GCs in the modulation of *SSTR5* expression, PAS showed in vitro antiproliferative and antisecretory effects mediated by high levels of *Sstr5* at different concentrations in the murine corticotroph pituitary tumor cell line AtT20 and in human corticotroph pituitary tumors (5, 27, 38, 39). In support of this evidence, clinical trials with PAS reported the normalization of urinary cortisol levels in 20% to 40% of patients with CD, with a better outcome observed in mild-moderate disease (6, 40, 41), although, to date, there are no studies correlating the clinical efficacy of PAS with SSTR5 expression in corticotroph pituitary tumors.

The current study demonstrates that combined treatment with OCT and miR-375 inhibitor significantly potentiates the antiproliferative effects of OCT in AtT20/D16 cell lines by increasing the bioavailability of SSTR2; in contrast to PAS, whose combination with miR-375 inhibitor does not affect its antiproliferative action in the murine cell model. These findings indicate that miR-375 can modulate OCT but not PAS response treatment in a model of corticotroph pituitary tumor. The lack of boosted efficacy of PAS could be explained by the different modulations in SSTRs trafficking induced by OCT and PAS. Indeed, despite the miR-375-dependent increased SSTR2 expression, a previous study has demonstrated that PAS is less potent than OCT in inducing internalization and signaling of SSTR2, by triggering rapid recycling of SSTR2 to the plasma membrane following endocytosis, indicating a lower internalization (42). This is mainly due to the different selective residues of SSTR2 phosphorylated, which accelerate receptor internalization (42).

Furthermore, intracellular localization has been taken into consideration to gain further insight into the role of miR-375 in modulating SSTR2 expression. Notably, the elevated protein expression of SSTR2 is also associated with a distinctive intracellular localization of the receptor itself. In particular, the current study demonstrates that miR-375 inhibition increases membranous SSTR2 expression, while its internalization was observed in the cytoplasm after OCT treatment, which appeared more pronounced when OCT treatment was administered in the presence of the miR-375 inhibitor. Although the functional assays have been performed on a murine pituitary corticotroph cell model, it is mandatory to point out that miRNAs are generally well-conserved across species, supporting the relevance of the findings of the current study. Additionally, although it was not feasible to perform activity assays on human cells, the ability of miR-375 in regulating SSTR2 expression has also been observed in 2 human pituitary corticotroph tumors.

Recent in vitro studies have demonstrated that treatment with OCT does not modify ERK1/2 phosphorylation in AtT20 cells, in contrast to PAS, which significantly reduces ERK1/2 phosphorylation (38). Conversely, miR-375 appears to be involved in the regulation of ERK1/2 phosphorylation. Indeed, further in vitro data demonstrated that mitogen-activated protein kinase kinase kinase-8 (MAP3K8), a serine-threonine kinase involved in turn in ERK phosphorylation, is a target gene of miR-375. Inhibition of miR-375 resulted in increased phosphorylation of both ERK1/2 and MAP3K8, suggesting a close relation between miR-375 and the MAPK pathway (14). The current study corroborates previous findings, demonstrating that OCT treatment alone does not affect ERK 1/2 phosphorylation. However, combined treatment with miR-375 inhibitor was found to increase phosphorylation levels of ERK 1/2, concomitantly with the observation of inhibition of cell proliferation and induction of apoptosis through the activation of caspase 3 and PARP cleavage.

In conclusion, the results of this study demonstrated that patients with CD exhibited significantly elevated circulating levels of miR-375 compared with healthy subjects. The overproduction of GCs, characterizing patients with CD, results in the upregulation of miR-375 and consequent downregulation of SSTR2 expression levels in corticotroph tumor tissue. The inhibition of miR-375 has been demonstrated to significantly increase SSTR2 protein expression, in particular its membranous localization, and to positively affect the antiproliferative properties of OCT. These findings suggest that miR-375 is a key regulator in the GC-dependent epigenetic modulation of SSTR2 expression and function.

## Data Availability

The datasets used and/or analyzed during the current study are available from the corresponding author upon reasonable request.

## Abbreviations

ACTH: adrenocorticotroph hormone
CD: Cushing disease
DAPI: 4.6-diamidino-2-phenylindole
DEX: dexamethasone
DMEM: Dulbecco’s modified Eagle’s medium
FBS: fetal bovine serum
GC: glucocorticoid
IF: immunofluorescence
OCT: octreotide
PAS: pasireotide
PBS: phosphate-buffered saline
RT-qPCR: reverse transcription quantitative polymerase chain reaction
SRL: somatostatin receptor ligand
SSTR: somatostatin receptor
WB: Western blot
